# Improving the Reporting Quality of Studies on Information Extraction From Clinical Texts: Protocol for the Development of a Consensus-Based Reporting Guideline

**DOI:** 10.2196/76776

**Published:** 2025-09-24

**Authors:** Daniel Reichenpfader, Henning Müller, Kerstin Denecke

**Affiliations:** 1 Institute for Patient-Centered Digital Health, Department of Engineering and Computer Science, Bern University of Applied Sciences, Quellgasse 21, Biel, 2502, Switzerland, 41 31 848 60 93; 2 PhD School of Life Sciences, Faculty of Medicine, University of Geneva, Geneva, Switzerland; 3 Department of Radiology and Medical Informatics, University of Geneva, Geneva, Switzerland; 4 Informatics Institute, HES-SO Valais-Wallis, Sierre, Switzerland

**Keywords:** reporting guideline, information extraction, artificial intelligence, natural language processing, consensus-based approach, eDelphi study

## Abstract

**Background:**

Information extraction (IE) from clinical texts is increasingly important in health care; yet, reporting practices remain inconsistent. Existing guidelines do not fully address the unique challenges of IE studies. IE methods vary widely in their design, ranging from rule-based systems to advanced large language models, contributing to heterogeneity in reporting. While several reporting frameworks exist for applications of artificial intelligence in health care, they primarily focus on prediction modeling or clinical trials and associated protocols rather than text-based IE.

**Objective:**

This study aims to develop the Clinical Information Extraction (CINEX) guideline, a consensus-based reporting guideline for studies on clinical IE.

**Methods:**

The CINEX guideline is developed following an established guideline methodology, including a 3-round electronic Delphi (eDelphi) study with domain experts and a final in-person consensus meeting. The eDelphi process includes feedback loops and predefined consensus thresholds, with items rated on a 10-point scale for both relevance and maturity. The final consensus meeting is held as a hybrid workshop at the MEDINFO 2025 conference and focuses on finalizing the items that reached consensus.

**Results:**

Our results will provide a validated reporting guideline for studies on clinical IE. A preliminary set of 28 reporting items was drafted from a scoping review and existing frameworks. The draft guidelines include 5 key dimensions: information model, architecture, data, annotation, and outcome. This draft guideline will be refined through the eDelphi process. It is designed to be technology-agnostic and applicable across diverse IE approaches, including not only large language models but also traditional machine learning methods and rule-based and hybrid systems.

**Conclusions:**

The CINEX guideline provides structured, expert-validated guidance for reporting clinical IE studies, improving transparency, reproducibility, and comparability. The final guideline will be disseminated alongside an explanatory document to support adoption and implementation.

## Introduction

Information extraction (IE) refers to techniques that automatically identify and structure key information from unstructured text. These methods enable efficient reuse of clinical narratives, supporting decision-making, research, and automation in electronic health records. Specific use cases include phenotyping (eg, extraction of specific diseases), drug-related tasks (eg, dosage IE), and clinical workflow optimization (eg, adverse event detection) [1]. Furthermore, IE can reduce the burden of manual chart review, enable large-scale epidemiological studies, and support real-time decision support.

Recent advances in natural language processing (NLP), especially through large language models (LLMs), have improved IE capabilities. However, this rapid technical evolution has introduced fragmentation in methods, terminology, and evaluation. We identified this heterogeneity during a scoping review of studies describing IE specifically from radiology reports. Studies vary widely in defining the target information, annotating reference standards, evaluating system performance, and disclosing implementation details [2]. Without standardized reporting, it becomes difficult to interpret results, compare systems, replicate experiments, or translate the developed effective algorithms into clinical practice. This suboptimal reporting quality had already been identified in a systematic review conducted by Davidson et al [3] in 2021.

To address reporting variability in artificial intelligence (AI) studies, several guidelines have emerged. The CONSORT-AI (Consolidated Standards of Reporting Trials–Artificial Intelligence) extension provides a checklist for reporting clinical trials that include AI-based interventions [4]. Its counterpart, SPIRIT-AI (Standard Protocol Items: Recommendations for Interventional Trials–Artificial Intelligence), focuses on protocol reporting for such trials, ensuring clarity and completeness before trial execution [5]. More recently, TRIPOD+AI (Transparent Reporting of a multivariable prediction model for Individual Prognosis Or Diagnosis–Artificial Intelligence) [6] and TRIPOD-LLM (Transparent Reporting of a Multivariable Prediction Model for Individual Prognosis Or Diagnosis specifically tailored for large language models) [7] have extended the original TRIPOD (Transparent Reporting of a Multivariable Prediction Model for Individual Prognosis Or Diagnosis) [8] guidelines to include the application of regression and machine learning methods as well as LLM-based approaches for prediction model studies. These guidelines promote transparency and reproducibility for studies describing AI-based interventions and prediction models. However, none are applicable to the reporting needs of IE from clinical text, which involves distinct tasks, input-output structures, and evaluation methods as compared to diagnostic modeling or intervention and their associated trials.

This paper presents the protocol for the development of the Clinical Information Extraction (CINEX) guideline, a consensus-based reporting guideline for clinical IE studies. This protocol outlines the methodology behind the CINEX guideline, including a 3-round modified electronic Delphi (eDelphi) process with domain experts. The development of this guideline follows best practices for health research reporting and includes steps for item refinement, a final in-person consensus meeting, and the publication of an explanation and elaboration (E&E) document. The CINEX guideline aims to close the reporting gap in clinical IE research, promoting rigor, transparency, and harmonization across diverse methodological paradigms.

## Methods

### Overview and Preliminary Guideline Development

The planned development process of the CINEX guideline was registered and publicly listed with the EQUATOR (Enhancing the Quality and Transparency Of Health Research) Network as a reporting guideline under development in August 2024 prior to its official start in October 2024 [9]. The EQUATOR Network is an international initiative that promotes transparent and accurate reporting of health research to improve the reliability and value of the published literature [10]. The CINEX guideline follows the methodological guidance outlined by Moher et al [11] for developing health research reporting guidelines. Throughout this protocol, references to Moher’s checklist items are indicated in angle brackets (< >).

The need for this guideline was identified during a scoping review of studies on LLM-based IE from radiology reports <1> [2]. The review aimed to assess the current state of the art in terms of performance, training and modeling approaches, clinical use cases, datasets, annotation methods, and commonly reported challenges. Derived from the review’s aims, a data extraction table was drafted and populated by one author. The scoping review revealed important barriers to study comparability, including untransparent performance metric calculations, lack of external validation, and limited availability of source code. These problems hinder transparency and reproducibility in the field. Based on the data extraction table as well as inspired by existing reporting guidelines, an initial set of 28 candidate reporting items was drafted and published <2> [12].

### eDelphi Study

To refine the preliminary guideline, a 3-round eDelphi study was planned between May and July 2025 <5,6>. The Delphi method was chosen as a structured, iterative process to achieve expert consensus, in line with the recommendations of Moher et al [11] for reporting guideline development.

We designed the eDelphi process based on the methodological principles of Häder [13], Nasa et al [14], as well as Trevelyan and Robinson [15]. We aim to recruit between 20 and 30 participants <4>. Minimum response rates are set at 30% for the first round and 70% for the following rounds, consistent with Häder’s [13] guidance.

Eligible participants include the authors of studies identified in the preceding scoping review, as well as domain experts with regard to clinical IE, recruited through the personal and professional networks of the executive committee. Interested individuals will be invited via email to join the study. The survey itself will be conducted using the open-source tool LimeSurvey [16].

The eDelphi study will comprise exactly 3 rounds [13]. Consensus for the inclusion of an item in the guideline will be defined as a mean rating of 8 or higher on a 10-point scale, with an SD of 2 or less. Exclusion will be defined as a mean rating of 3 or lower, also with an SD of ≤2. Items falling outside these thresholds will be revisited during a final consensus meeting held after the eDelphi process. Response stability is recorded but not used to assess early termination. Only in the first round, panelists have the possibility to add additional items for each domain. Exclusion as well as finalization due to consensus of items is only conducted after rounds 2 and 3. Rephrasing of items and descriptions is conducted after each round based on the panelists’ comments.

In the first round, participants will be presented with the draft reporting items, including any proposed value sets where applicable. They will be asked to assess the relevance and maturity of each item using a 10-point scale with labeled endpoints only (“not at all relevant” and “not at all mature” to “very relevant” and “very mature”). Each item additionally includes a default “No answer” option. To approximate interval-level data, a larger number of anchor points was selected instead of a classical 5-point scale based on methodological recommendations [17]. To exclude or include (finalize) an item, the abovementioned mean and SD thresholds must be met for both relevance and maturity.

Feedback will be proactively provided by the executive committee for each new round (active feedback loop): participants re-evaluate items that were not excluded, assisted by aggregated ratings of the whole panel (as histograms), moderated anonymized comments from the previous round, and their own previous ratings. This format enables reflection and informed re-evaluation.

Besides participating in the eDelphi panel, each expert completes a self-disclosure of their expertise in clinical IE. Consistent with Häder [13], no demographic data will be collected beyond gender and country of affiliation. Anonymity among participants will be preserved throughout the eDelphi panel. At the end of the first round, participants will be invited to indicate whether they wish to participate in the final consensus meeting taking place after the completion of the third round and to contribute to the final publication of the CINEX guideline and the explanatory document.

A pretest of the first-round survey will be conducted among the executive committee (comprising authors DR, HM, and KD) to ensure clarity and functionality. These individuals will not participate in the actual eDelphi rounds.

### Consensus Meeting, Finalization, and Outlook

For the final step of the guideline development process, a face-to-face hybrid consensus meeting is conducted, organized as a workshop at the MEDINFO conference in August 2025 <6,7,8>. During the workshop, the results of the Delphi process are presented, and items that achieved consensus are reviewed for resolving minor ambiguities and final phrasing. Items without prior agreement are discussed and, if needed, resolved by open voting. Consensus is defined as ≥80% agreement. The meeting is considered quorate if ≥50% of the panelists of the first round of the eDelphi panel are present. Persistent disagreements will be documented and reported transparently in the publication of the final guideline. The workshop shall result in the formal finalization of the CINEX guideline; thereafter, the guidance statement is finalized <9>, complemented by an E&E document <10>, and both are made available as publication <11>. The guideline development process will be reported in a structured way in accordance with the Accurate Consensus Reporting Document (ACCORD) reporting guideline [18].

Responsibilities for postpublication activities <12‐18> are divided among the contributing authors: the CINEX guideline will be hosted on an open access online platform for ongoing feedback <12,14,16>, will be pilot-tested by panelists, and its impact on reporting quality will be evaluated. To ensure durability, an executive group will review the CINEX guideline at regular 3-year intervals, evaluating its impact <15> and issuing updates when new evidence or methods make a revision necessary <18>. We seek endorsement from journals and societies <13>. Translation is not planned for the initial release <17>. By following these postpublication activities, we aim at establishing a long-term relevance of the CINEX guideline.

### Ethical Considerations

This study was granted an exemption by the competent ethics committee of the canton of Bern on April 29, 2025 (ID: Req-2025‐00587). All eDelphi study participants gave electronic informed consent prior to participation.

## Results

A preliminary guideline comprising 28 items and 5 dimensions (information model, architecture, data, annotation, and outcome) was published in August 2024 [12]. These items will be presented in the first round of the eDelphi study.

## Discussion

### Principal Results

This protocol presents the development of the CINEX guideline, a reporting guideline specifically developed for clinical IE studies. The CINEX guideline addresses key challenges in the field—including inconsistent terminology, reporting, and evaluation—by providing a structured, technology-agnostic framework tailored to IE tasks in health care. By design, the CINEX guideline accommodates both rule-based and data-driven approaches, including LLM-based methods by focusing on essential reporting dimensions (eg, data sources, annotation strategies, information model, and evaluation metrics) that apply across paradigms. This ensures that differences in implementation, such as rule-based extraction pipelines or end-to-end architectures, can be transparently described and compared within a shared reporting structure. To facilitate adoption, we will pursue parallel endorsement of the CINEX guideline by multiple journals in biomedical informatics, promote its use by providing an online website (inspired by TRIPOD-LLM [7]), and invite panelists to apply the CINEX guideline in their own research.

### Limitations

Our approach has several limitations. First, the development process for the CINEX guideline is based on guidance for reporting guideline development in health research as outlined by Moher et al [11]. While the CINEX guideline addresses health-related research, it also intersects significantly with the computer science domain. Currently, however, there are no methodological frameworks for reporting guideline development in computer science. Given this gap, we adopted the framework of Moher et al [11], which is well-established and has informed the development of most reporting guidelines endorsed by the EQUATOR Network, including guidelines with a technical focus (eg, CONSORT-AI). Nonetheless, we acknowledge that this choice may not fully capture disciplinary nuances outside the health sciences. The CINEX guideline will therefore be developed with input from the computer sciences as well as clinical disciplines and be tested for clarity and relevance in both contexts.

Participant recruitment relies partly on professional networks, which may limit diversity. While consensus thresholds and item rating methods are clearly defined a priori with this protocol, they remain subjective to a certain degree. The final in-person consensus meeting, although valuable for discussion, may introduce social influence and new opinions from participants that have not participated in the eDelphi panel. Additionally, the CINEX may require future updates to accommodate rapid developments in LLMs and multimodal approaches.

### Comparison With Prior Work

The CINEX guideline builds on the methods used in guidelines like CONSORT-AI, SPIRIT-AI, and TRIPOD-LLM and is the first to focus specifically on clinical IE. Our study design incorporated detailed feedback loops and a 10-point scale instead of a 5-point scale aiming to improve rating precision. While existing AI guidelines address broader study types, none are tailored to the unique needs of clinical text-based IE, underscoring the CINEX guideline’s distinct contribution. Furthermore, we seek to address the challenges faced by prior reporting guidelines: first, adoption and enforcement by journals have often been inconsistent, thus limiting their impact; to mitigate this, we will accompany the CINEX guideline with an online template checklist for authors and actively engage journals to encourage endorsement. Second, ambiguity in checklist items has been a barrier to compliance; therefore, the CINEX guideline will be supported by a detailed E&E document. Third, the CINEX guideline will include example items tailored for computer science as well as medical audiences, supporting broader adoption across disciplines.

### Conclusions

The CINEX guideline fills a critical gap in the reporting of clinical IE studies by offering a dedicated, consensus-driven framework. It aims to improve reproducibility, comparability, and transparency in this growing area of clinical NLP. Continued community involvement and iterative updates will be essential to ensure its ongoing relevance.

## Abbreviations

ACCORD: Accurate Consensus Reporting Document
AI: artificial intelligence
CINEX: Clinical Information Extraction
CONSORT-AI: Consolidated Standards of Reporting Trials–Artificial Intelligence
E&E: explanation and elaboration
eDelphi: electronic Delphi
EQUATOR: Enhancing the Quality and Transparency Of Health Research
IE: information extraction
LLM: large language model
NLP: natural language processing
SPIRIT-AI: Standard Protocol Items: Recommendations for Interventional Trials–Artificial Intelligence
TRIPOD+AI: Transparent Reporting of a multivariable prediction model for Individual Prognosis Or Diagnosis–Artificial Intelligence
TRPIOD-LLM: Transparent Reporting of a Multivariable Prediction Model for Individual Prognosis Or Diagnosis specifically tailored for large language models
